# Systemic Review and Meta-Analysis of the Clinical Efficacy and Adverse Effects of Zhengqing Fengtongning Combined with Methotrexate in Rheumatoid Arthritis

**DOI:** 10.1155/2015/910376

**Published:** 2015-08-24

**Authors:** Xiu-min Chen, Run-yue Huang, Qing-chun Huang, Yong-liang Chu, Jing-yao Yan

**Affiliations:** ^1^Department of Rheumatology, The Second Affiliated Hospital, Guangzhou University of Chinese Medicine, Guangzhou, Guangdong, China; ^2^Postdoctoral Mobile Research Station, Guangzhou University of Chinese Medicine, Guangzhou, Guangdong, China

## Abstract

Chinese medicines are gaining wider acceptance. They have been used for treating rheumatoid arthritis (RA) for thousands of years, and the need to investigate the interaction between Chinese medicines and western medicines is widely recognized. In this study, a large number of RCTs and CCTs were analyzed to systematically assess the effects and adverse events of Zhengqing Fengtongning (ZQFTN) for RA. Eleven studies that contained 956 participants (508 in the treatment group; 448 in the control group) were included. The results showed that although ZQFTN combined with methotrexate MTX could not decrease the swollen joint count and tender joint count of RA patients better than MTX alone, the combination therapy might relieve the duration of morning stiffness (SMD: −16.06; 95% CI: −28.77 to −3.34), reduce laboratory indexes (RF: SMD: −10.84; 95% CI: −19.39 to −2.29; ESR: SMD: −7.26; 95% CI: −11.54 to −2.99; CRP: SMD: −3.66; 95% CI: −5.94 to −1.38), and improve the overall effect (RR: 1.08; CI: 1.01 to 1.16) better than monotherapy. The combination therapy was significantly better in controlling adverse drug reactions (RR: 0.60; 95% CI: 0.46 to 0.79). Through this systematic review, we found that ZQFTN combined with MTX for the treatment of RA might have better clinical efficacy than MTX only and might be superior in terms of controlling adverse drug reactions.

## 1. Introduction

Rheumatoid arthritis (RA) is a chronic systemic inflammatory disease, characterized by inflammation of the synovial tissue and damage to articular cartilage and bone leading to severe disability, functional decline, and accelerated mortality [1, 2]. RA is the most common inflammatory rheumatic disease, with a prevalence of 0.5%–1.0% in Europe and North America. In Southeast Asia, including Japan and China, the prevalence is slightly lower, accounting for 0.2%-0.3% [3]. In China, up to 5 million people suffer from RA [4], which imposes a considerable burden on patients, their families, and society. Treatment options for RA include nonsteroidal anti-inflammatory drugs (NSAIDs), disease-modifying antirheumatic drugs (DMARDs), local steroids, and biologics. Methotrexate (MTX), one of the DMARDs, is the first-line drug for treating RA [5]. The treatment methods do not retard or stop the radiographic progression or prevent joint damage. Some studies have demonstrated that 30% of the patients in remission had radiographic progression, 96% had synovitis, and 35% had osteitis [6, 7]. Drug toxicity, high costs, and lack of long-term safety data are, to some extent, inevitable problems for application of the current therapeutic strategies. These factors lead to the use of complementary and alternative medicines (CAMs), including meditation, acupuncture, chiropractic, vitamin and mineral therapy, herbal medicine, and hypnotherapy.

Alternatively, Chinese medicine could be a viable treatment option because it has been used to treat human diseases in China and other parts of the world for thousands of years. Chinese medicine could effectively treat RA with a low adverse reaction [19]. Sinomenine (SIN) was isolated from* Sinomenium acutum*, a Chinese medicinal plant that has been used for treating rheumatoid diseases including RA for over 2000 years [20, 21]. SIN has a variety of pharmacological effects including analgesia, anti-inflammatory properties, and immune suppression [22]. Zhengqing Fengtongning (ZQFTN), as a SIN preparation, has been used for treating RA for many years. As ZQFTN is used with increasing frequency, the need to investigate the interactions between ZQFTN and western drugs is widely recognized. In this paper, we performed a systematic review with meta-analysis of randomized controlled trials that compared ZQFTN in combination with MTX with MTX monotherapy. The objective of this study was to determine whether ZQFTN combined with MTX is superior and safer than MTX monotherapy for treating RA. We hypothesized that the results of this systematic review could summarize the available evidence for clinicians to RA treatment using SIN preparations.

## 2. Materials and Methods

This systematic review and meta-analysis followed the Preferred Reporting Items for Systematic Reviews and Meta-Analyses (PRISMA) statement to ensure its accuracy [23].

### 2.1. Search Strategy

A systematic review was conducted by searching multiple databases including Medline/PubMed, Embase, the China National Knowledge Infrastructure (CNKI), the Chinese Medical Journal Database, Wanfang Data, VIP, the Chinese Biomedical Literature Database, the Chinese Scientific and Technological Journals Database, the Traditional Chinese Medicine Database, the China Doctorate Dissertation Full Text Database, and other databases. The databases were searched from their start date to December 2013. For the English databases, free text terms such as “Sinomenine” or “*Sinomenium*” or “Zhengqing Fengtongning” and “rheumatoid arthritis” or “RA” were used. For the Chinese databases, free text terms such as “qing feng teng” or “qing teng jian” or “Zhengqing Fengtongning” and “lei feng shi guan jie yan” or “pain paralysis” were used. The languages were limited to English or Chinese. A secondary search was conducted, and ambiguous literature was searched as well.

### 2.2. Selection Criteria

Randomized controlled trials (RCTs) and controlled clinical trials (CCTs) that involved the efficacy and/or safety of ZQFTN to treat RA were selected. The studies were selected for analysis if they satisfied the following criteria. (1) The subjects took ZQFTN combined with MTX versus MTX only. (2) The included participants were patients with a clear diagnosis of RA. The diagnostic criteria for RA in the trials accorded with the American Rheumatism Association 1987 revised criteria for the classification of RA [24]. (3) ZQFTN was used as an active treatment intervention. (4) There were no restrictions regarding sex, age, severity, and duration of RA.

### 2.3. Outcome Assessment

The outcome measures included enumeration data and measurement data. The enumeration data pertained to the clinical efficacy and adverse events. The number of patients treated effectively in each group was counted based on the categories of cured, markedly effective, and effective. The measurement data included rheumatoid factor (RF), C-reactive protein (CRP), erythrocyte sedimentation rate (ESR), tender joint count (TJC), swollen joint count (SJC), and duration of morning stiffness (DMS).

### 2.4. Data Extraction

Three authors participated in the data extraction of all the studies included in the review. Two authors (Xiumin Chen and Runyue Huang) first extracted the relevant data including the first author, publication year, total number of cases included in the experimental group (EG) and the control group (CG), intervention methods, and endpoint evaluation indicators, independently. Disagreements were resolved by consensus or were arbitrated by the third author (Qingchun Huang).

### 2.5. Quality Assessment of the Included Studies

The quality of each study included in this review was assessed using the Cochrane Handbook for Systematic Reviews of Interventions and Jadad scoring [25]. The details that were assessed were as follows: (1) whether the test methods were random, (2) whether allocation concealment was achieved, (3) whether blinded tests were adopted, and (4) whether patients were lost because of follow-up or quitting. Scoring of 1–3 indicated low quality, whereas 4–7 indicated high quality [26].

### 2.6. Statistical Methods

Review Manager (Revman) (Computer program), version 5.3. (The Nordic Cochrane Centre, The Cochrane Collaboration, Copenhagen, 2014), was used to analyze the collected clinical research data. The enumeration data were evaluated using the relative risk (RR) and 95% confidence interval (CI), and the measurement data were combined using the standardized mean difference (SMD) and 95% CI. Analysis was carried out using a fixed or random effects model according to the heterogeneity. The percentage of heterogeneity in the study was determined by the *I* ^2^ statistic, with a value of 0% indicating no heterogeneity and larger values indicating increased heterogeneity. A *P* value <0.10 was considered to suggest statistical heterogeneity and prompted random effects modelling.

## 3. Results

### 3.1. Literature Search Results

**Figure 1:**
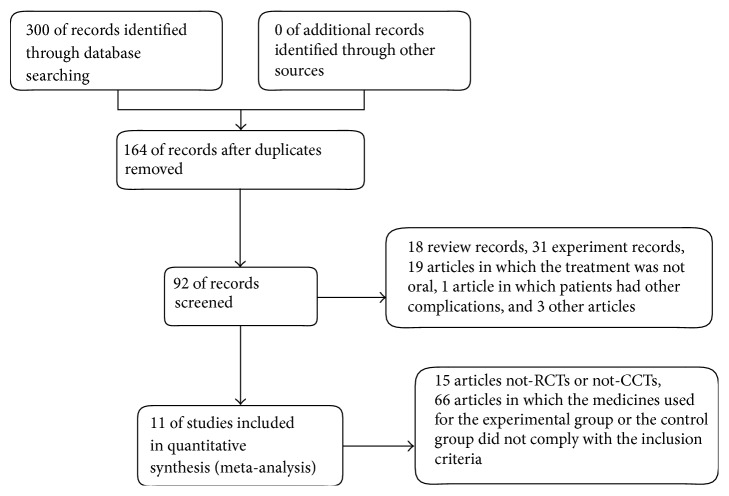
Process of searching and screening studies.

Using the search strategy, 300 studies were retrieved. After removal of duplicates across databases, 164 studies were screened. From the 164 studies, 11 studies that met the inclusion criteria were included in this study for the systematic review [8–18]. The study selection process is shown in Figure 1. The characteristics of the studies are summarized in Table 1. These studies included a total of 956 participants, 508 in the treatment group and 448 in control group. The duration of most studies was 12 or 24 weeks. The dose of MTX in the combination therapy groups ranged between 7.5 and 10 mg/week except for one study (15 mg/week) [10], while the dose was larger in the monotherapy groups, ranged between 10 and 15 mg/week. The doses of ZQFTN ranged between 60 and 240 mg/day but most were 120 mg/day.

### 3.2. Quality of Included Systematic Studies

**Figure 2:**
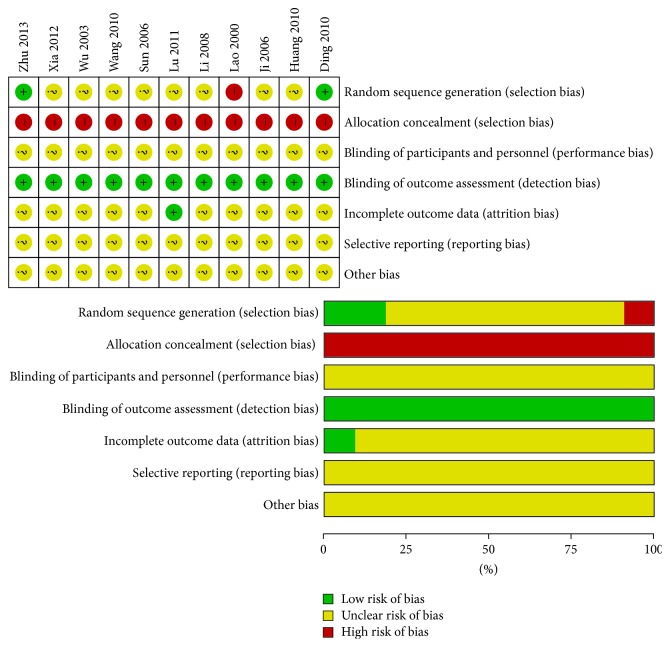
Risk of bias summary and risk of bias graph.

Most of the included studies were of low quality because of unclear randomization, inefficient allocation concealment, inadequate blinding, or described withdrawals and dropouts. The high-quality studies accounted for 27.27% (3/11) of the total studies. In addition, 90.91% (10/11) of the studies mentioned a random and blinded design, and only 18.18% (2/11) described the random design; 9.09% (1/11) described the dropouts, whereas 90.91% (10/11) of the studies failed to mention the number of patients who quit the study or were lost during follow-up (Figure 2).

### 3.3. Total Effect of ZQFTN

**Figure 3:**
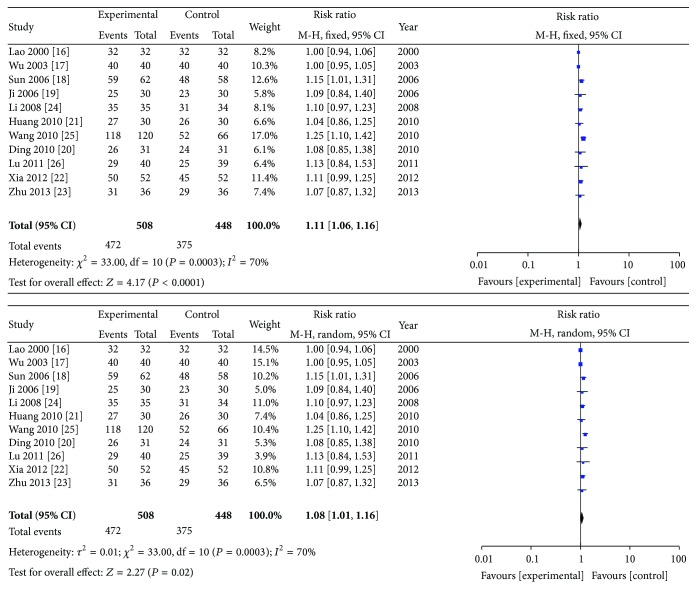
Analysis of the total effect of ZQFTN combined with MTX and MTX only for the treatment of RA ((1) study, first name of the first author, publishing year, and the number of studies; (2) experimental: the group of MTX combined with ZQFTN; control: the group of MTX only; (3) *I*-squared and *P* are the criterion of heterogeneity test; ◆: pooled relative risk; -■-: relative risk and 95 confidence interval).

The 11 studies were analyzed for comparisons of the total effect of ZQFTN combined with MTX and of MTX only. The data indicated that 472 patients (92.91%) improved after treatment with ZQFTN combined with MTX whereas 375 patients (83.71%) improved after treatment with MTX only. The meta-analysis was performed using a random effects model because of the high heterogeneity (*I* ^2^ = 70%, *P* < 0.10). The combined RR was 1.08, and the 95% CI was 1.01 to 1.16 (*P* = 0.02), indicating that ZQFTN combined with MTX was better in improving the overall symptoms of RA patients than MTX only (Figure 3).

### 3.4. Rheumatoid Factor (RF)

**Figure 4:**
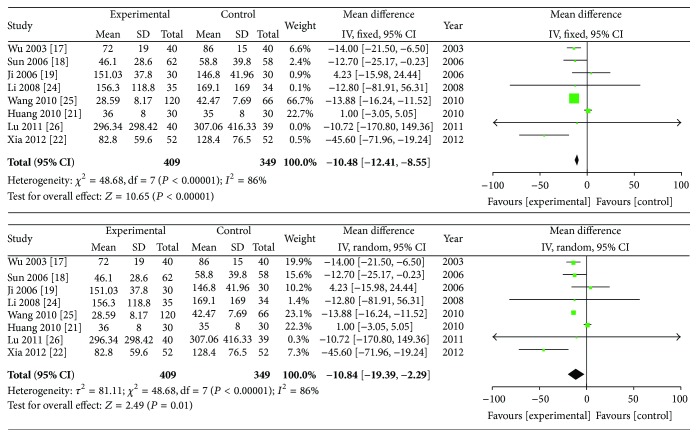
Analysis of RF of ZQFTN combined with MTX and MTX only for the treatment of RA ((1) study, first name of the first author, publishing year, and the number of studies; (2) experimental: the group of MTX combined with ZQFTN; control: the group of MTX only; (3) *I*-squared and *P* are the criterion of heterogeneity test; ◆: pooled relative risk; -■-: relative risk and 95 confidence interval).

Eight studies including 758 patients (409 in the experimental group and 349 in the control group) provided the serum RF concentration data. The *I*-squared was 86% and the *P* value was <0.10 indicating high heterogeneity, so a random effects model was adopted for the meta-analysis. The combined SMD was −10.84 with a 95% CI of −19.39 to −2.29 (*P* = 0.01). Therefore, ZQFTN combined with MTX and MTX showed significant differences in reducing the serum RF concentration in RA patients (Figure 4).

### 3.5. Erythrocyte Sedimentation Rate (ESR) (mm/h)

**Figure 5:**
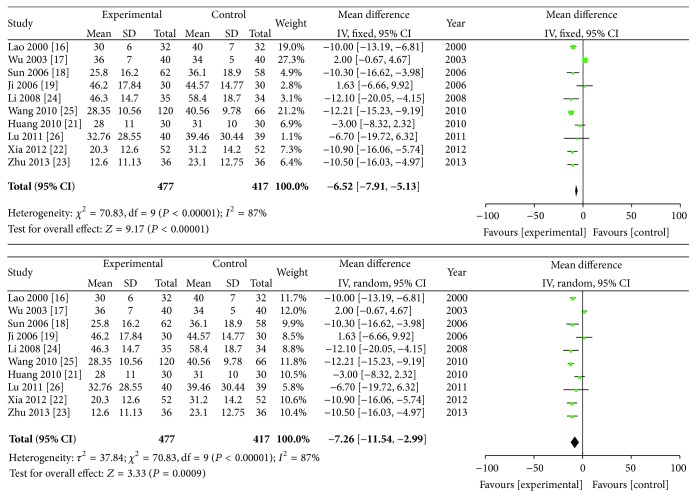
Analysis of ESR of ZQFTN combined with MTX and MTX only for the treatment of RA ((1) study, first name of the first author, publishing year, and the number of studies; (2) experimental: the group of MTX combined with ZQFTN; control: the group of MTX only; (3) *I*-squared and *P* are the criterion of heterogeneity test; ◆: pooled relative risk; -■-: relative risk and 95 confidence interval).

Ten studies provided ESR data, and a random effects model was used for the analysis. The combined SMD was −7.26, and the 95% CI ranged from −11.54 to −2.99 (*P* < 0.01). There was an obvious difference between the effects of ZQFTN combined with MTX and of MTX alone in reducing the ESR (Figure 5).

### 3.6. C-Reactive Protein (mg/L)

**Figure 6:**
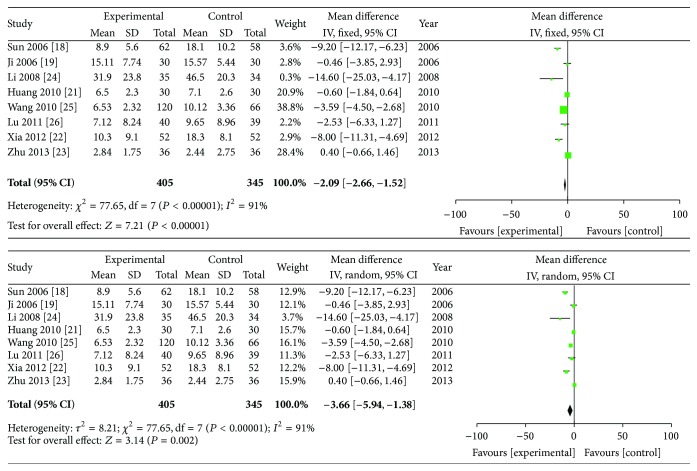
Analysis of CRP of ZQFTN combined with MTX and MTX only for the treatment of RA ((1) study, first name of the first author, publishing year, and the number of studies; (2) experimental: the group of MTX combined with ZQFTN; control: the group of MTX only; (3) *I*-squared and *P* are the criterion of heterogeneity test; ◆: pooled relative risk; -■-: relative risk and 95 confidence interval).

Eight studies provided the C-reactive protein data. The meta-analysis showed that *I* ^2^ = 93% which meant high heterogeneity and the analysis was performed using a random effects model. The combined SMD was −3.66, and the 95% CI ranges from −5.94 to −1.38 (*P* < 0.01). Significant differences were found in the reduction of serum CRP levels between the group of ZQFTN combined with MTX and the MTX group (Figure 6).

### 3.7. Duration of Morning Stiffness, Swollen Joint Count, and Tender Joint Count

**Figure 7:**
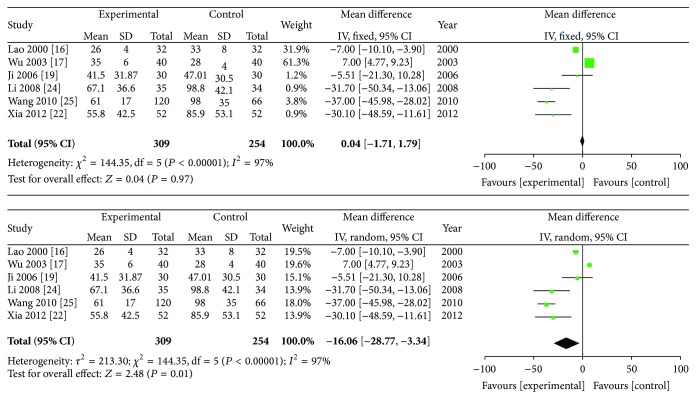
Analysis of duration of morning stiffness of ZQFTN combined with MTX and MTX only for the treatment of RA ((1) study, first name of the first author, publishing year, and the number of studies; (2) experimental: the group of MTX combined with ZQFTN; control: the group of MTX only; (3) *I*-squared and *P* are the criterion of heterogeneity test; ◆: pooled relative risk; -■-: relative risk and 95 confidence interval).

**Figure 8:**
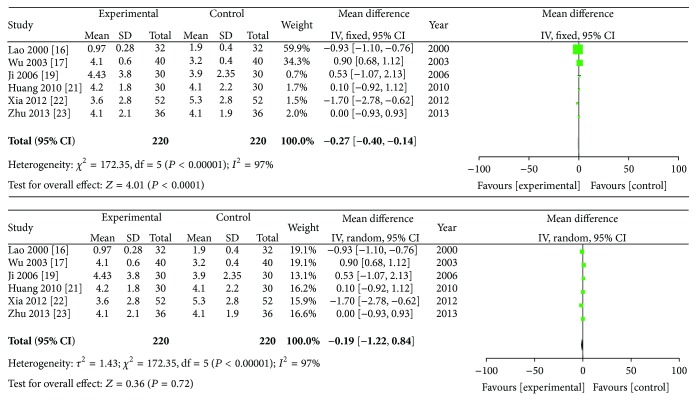
Analysis of swollen joint count of ZQFTN combined with MTX and MTX only for the treatment of RA ((1) study, first name of the first author, publishing year, and the number of studies; (2) experimental: the group of MTX combined with ZQFTN; control: the group of MTX only; (3) *I*-squared and *P* are the criterion of heterogeneity test; ◆: pooled relative risk; -■-: relative risk and 95 confidence interval).

**Figure 9:**
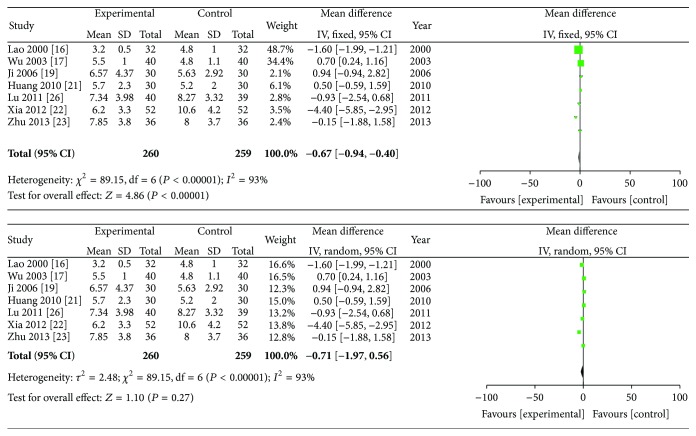
Analysis of tender joint count of ZQFTN combined with MTX and MTX only for the treatment of RA ((1) study, first name of the first author, publishing year, and the number of studies; (2) experimental: the group of MTX combined with ZQFTN; control: the group of MTX only; (3) *I*-squared and *P* are the criterion of heterogeneity test; ◆: pooled relative risk; -■-: relative risk and 95 confidence interval).

The duration of morning stiffness, swollen joint count, and tender joint count were analyzed in this review, and random effects models were adopted for the three analyses.  Figure 7 shows the results of the meta-analyses on the duration of morning stiffness. The data indicated that ZQFTN combined with MTX and MTX only showed significant differences in their ability to reduce the duration of morning stiffness in RA patients (SMD: −16.06; 95% CI: −28.77 to −3.34; *P* = 0.01).  Figure 8 shows the result of the meta-analysis on swollen joint count. The combined SMD was −0.19, and the 95% CI ranged from −1.22 to 0.84 (*P* = 0.72), indicating that there were no statistical significances between the effects of ZQFTN combined with MTX and MTX alone on reducing the swollen joint count.  Figure 9 presents the results of the meta-analyses on tender joint count. It shows that no differences were found on the ability of reducing the tender joint count between the group of ZQFTN combined with MTX and the MTX group (SMD: −0.71; 95% CI: −1.97 to −0.56; *P* = 0.27).

### 3.8. Adverse Effects (AEs)

**Figure 10:**
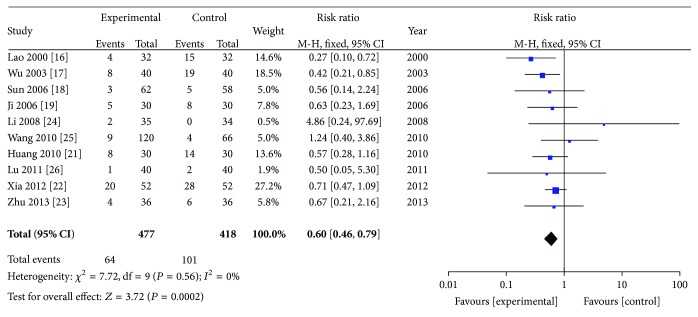
Analysis of adverse effects of ZQFTN combined with MTX and MTX only for the treatment of RA ((1) study, first name of the first author, publishing year, and the number of studies; (2) experimental: the group of MTX combined with ZQFTN; control: the group of MTX only; (3) *I*-squared and *P* are the criterion of heterogeneity test; ◆: pooled relative risk; -■-: relative risk and 95 confidence interval).

Ten studies provided AEs, including 64 patients (13.42%) in the experimental group and 101 (24.16%) in the control group. The analysis was performed using a fixed effects model because there had been no heterogeneity (*I* ^2^ = 0%). The results show that there were fewer adverse reactions overall when using ZQFTN combined with MTX to treat RA than when using MTX alone (RR: 0.60; 95% CI: 0.46 to 0.79; *P* < 0.01) (Figure 10).

## 4. Discussion

Although one systematic review and meta-analysis regarding the efficacy and safety of SIN in the treatment of RA have been reported, the systematic review is dated and it examined SIN versus NSAIDs for the treatment of RA [27]. It concluded that SIN preparations might possess an efficacy comparable to that of NSAIDs for ameliorating patients' major symptoms or signs as well as laboratory markers [27]. Nevertheless, as a therapeutic agent of RA, SIN could inhibit the development and progression of CIA in rats [28], might inhibit bFGF-induced angiogenesis in vitro and in vivo [29], could induce the apoptosis of macrophages through activation of the ERK pathway, and inhibit proliferation and induce apoptosis via activation of caspase 3 of CD4^+^ T cells [30]. In recent years, studies showed that the imbalance of T helper 17 (Th17)/T regulatory (Treg) cells played a crucial role in RA [31, 32], while SIN could regulate the balance of Th17/Treg cells in arthritis rats [32]. Therefore, SIN may be as herb DMARDs because of its immunomodulatory and anti-inflammatory activities to treat RA combined with MTX. Many regimens on SIN combined with MTX in the treatment of RA have been studied, but the relative benefit and toxicity of SIN combined with MTX versus monotherapy are not clear.

In our study, the efficacy and safety of ZQFTN combined with MTX versus MTX only for treating RA were reviewed. According to this study, 11 studies met the diagnosis as well as the inclusion and exclusion criteria, including 956 patients (508 in the treatment group and 448 in the control group). Revman 5.3 software was used for the data syntheses and meta-analyses. The results showed that ZQFTN combined with MTX had a better total effect than MTX alone. A statistically significant difference was shown in the reduction of the serum RF, ESR, and CRP levels of the RA patients between the 2 groups. The result in Figure 7 indicates that the combination therapy had a better effect than the monotherapy on reducing the clinical symptoms of morning stiffness duration. However, the ZQFTN groups and MTX groups showed a similar effect in the reduced numbers of swollen joints and tender joints in the patients with RA. Although ZQFTN combined with MTX could not decrease the swollen joint count and tender joint count of RA patients more than MTX only, the combination therapy might decrease the duration of morning stiffness, reduce the laboratory indexes, and improve the overall effect more effectively than monotherapy. Table 1 shows that the dose of MTX in combination therapy is smaller than that in monotherapy in most of the studies, indicating that combination therapy may reduce the dose of synthetic DMARDs.

The meta-analysis showed that there was a statistically significant difference in the total AEs. The most common AEs of the two groups were gastrointestinal discomfort, abnormal liver function, skin discomfort, blood cell reduction, menstrual disorders, dizziness, and amenorrhea. These AEs could be relieved with or without treatment and there were no withdrawals due to AEs.

The limitations of this meta-analysis should be noted. All of the included studies described above were performed in China because standardized ZQFTN was not available outside of China, and the basic and clinical effects of ZQFTN in other countries might not be consistent with these data. Many of the studies included a small number of patients. The journals in which these studies were published are of poor quality. The most important criteria, including ACR 20, ACR 50, and ACR 70, were not reported in all of the studies. Accordingly, the conclusions of this review should be carefully interpreted. Because of the increasing use of Chinese medicines, accurate data on the interactions between Chinese medicines and western medicines are required.

## 5. Conclusion

Through a systematic review of the clinical efficacy and safety of ZQFTN combined with MTX versus MTX only for the treatment of RA, we found that ZQFTN combined with MTX might have better clinical efficacy than MTX only for the treatment of RA. A small dose ZQFTN combined with MTX was superior to MTX alone for controlling adverse drug reactions. ZQFTN, as a type of herbal DMARD, appears to have higher effects and lower side effects than synthetic DMARDs. Considering the low methodological quality of the randomized trials, more RCTs are needed before ZQFTN could be recommended to replace or be combined with synthetic DMARDs.

## Figures and Tables

**Table 1:** Characteristics of the included studies.

Author, year	Sample size		Intervention methods		Duration	Outcomes
	EG	CG	EG	CG	(wks)	
Lao 2000 [8]	32	32	ZQFTN 20–40 mg tid, MTX 7.5 mg qw	MTX 15 mg qw	12	TE, TJC, SJC, DMS, RF, ESR, AE

Wu 2003 [9]	40	40	ZQFTN 40 mg tid, MTX 7.5 mg qw	MTX 15 mg qw	12	TE, TJC, SJC, DMS, RF, ESR, AE

Sun and Lan 2006 [10]	62	58	ZQFTN 120 mg qd, MTX 15 mg qw	MTX 15 mg qw	24	TE, RF, ESR, CRP, AE

Ji and Zhu 2006 [11]	30	30	ZQFTN 20–40 mg tid, MTX 7.5 mg qw	MTX 15 mg qw	12	TE, TJC, SJC, DMS, RF, ESR, CRP, AE

Ding 2010 [12]	31	31	ZQFTN 20–40 mg tid, MTX 7.5 mg qw	MTX 15 mg qw	12	TE

Huang and Wang 2010 [13]	30	30	ZQFTN 60 mg bid, MTX 7.5 mg qw	MTX 15 mg qw	12	TE, TJC, SJC, DMS, RF, ESR, CRP, AE

Xia et al. 2012 [14]	52	52	ZQFTN 60 mg tid, MTX 10 mg qw	MTX 10 mg qw	12	TE, TJC, SJC, DMS, RF, ESR, CRP, AE

Zhu et al. 2013 [15]	36	36	ZQFTN 60 mg bid, MTX 10 mg qw	MTX 10 mg qw	24	TE, TJC, SJC, DMS, ESR, CRP, AE

Li et al. 2008 [16]	35	34	ZQFTN 120 mg bid, MTX 10 mg qw	MTX 15 mg qw	8	TE, DMS, RF, ESR, CRP, AE

Wang 2010 [17]	120	66	ZQFTN 120 mg bid, MTX 10 mg qw	MTX 10 mg qw	24	TE, DMS, RF, ESR, CRP, AE

Lu and Su 2011 [18]	40	39	ZQFTN 120 mg bid, MTX 10 mg qw	MTX 10 mg qw	12	TE, TJC, RF, ESR, CRP, AE

Note: ZQFTN: Zhengqing Fengtongning; EG: experimental group; CG: control group; TE: total effect; TJC: tender joint count; SJC: swollen joint count; DMS: duration of morning stiffness; RF: rheumatoid factor; ESR: erythrocyte sedimentation rate; CRP: C-reactive protein; AE: adverse effect.
